# A Versatile Continuous Fluorometric Enzymatic Assay for Targeting Nicotinate Phosphoribosyltransferase

**DOI:** 10.3390/molecules28030961

**Published:** 2023-01-18

**Authors:** Gabriele Minazzato, Elisa Marangoni, Carlo Fortunato, Riccardo Petrelli, Loredana Cappellacci, Fabio Del Bello, Leonardo Sorci, Massimiliano Gasparrini, Francesco Piacente, Santina Bruzzone, Nadia Raffaelli

**Affiliations:** 1 Department of Agriculture, Food and Environmental Sciences, Polytechnic University of Marche, 60131 Ancona, Italy; 2 School of Pharmacy, Chemistry Interdisciplinary Project (ChIP), University of Camerino, 62032 Camerino, Italy; 3 Division of Bioinformatics and Biochemistry, Department of Materials, Environmental Sciences and Urban Planning, Polytechnic University of Marche, 60131 Ancona, Italy; 4 Department of Experimental Medicine, University of Genoa, 16126 Genoa, Italy

**Keywords:** NAD^+^ biosynthesis, NAPRT, enzyme activity assay

## Abstract

The maintenance of a proper NAD^+^ pool is essential for cell survival, and tumor cells are particularly sensitive to changes in coenzyme levels. In this view, the inhibition of NAD^+^ biosynthesis is considered a promising therapeutic approach. Current research is mostly focused on targeting the enzymes nicotinamide phosphoribosyltransferase (NAMPT) and nicotinate phosphoribosyltransferase (NAPRT), which regulate NAD^+^ biosynthesis from nicotinamide and nicotinic acid, respectively. In several types of cancer cells, both enzymes are relevant for NAD^+^ biosynthesis, with NAPRT being responsible for cell resistance to NAMPT inhibition. While potent NAMPT inhibitors have been developed, only a few weak NAPRT inhibitors have been identified so far, essentially due to the lack of an easy and fast screening assay. Here we present a continuous coupled fluorometric assay whereby the product of the NAPRT-catalyzed reaction is enzymatically converted to NADH, and NADH formation is measured fluorometrically. The assay can be adapted to screen compounds that interfere with NADH excitation and emission wavelengths by coupling NADH formation to the cycling reduction of resazurin to resorufin, which is monitored at longer wavelengths. The assay system was validated by confirming the inhibitory effect of some NA-related compounds on purified human recombinant NAPRT. In particular, 2-hydroxynicotinic acid, 2-amminonicotinic acid, 2-fluoronicotinic acid, pyrazine-2-carboxylic acid, and salicylic acid were confirmed as NAPRT inhibitors, with Ki ranging from 149 to 348 µM. Both 2-hydroxynicotinic acid and pyrazine-2-carboxylic acid were found to sensitize OVCAR-5 cells to the NAMPT inhibitor FK866 by decreasing viability and intracellular NAD^+^ levels.

## 1. Introduction

Nicotinamide adenine dinucleotide (NAD^+^) is an important cofactor in redox reactions of energetic metabolism and a consumable substrate of enzymes regulating key cellular processes, including differentiation, metabolic adaptation, inflammatory response, and signal transduction. NAD^+^ regeneration is guaranteed by multiple biosynthetic pathways that start from different precursors, namely tryptophan and vitamin B3, which comprises nicotinamide (NAM), nicotinic acid (NA), and nicotinamide riboside (NR). Therefore, four major different biosynthetic pathways contribute to the overall NAD^+^ pool. They can occur in different combinations and with different efficiencies, depending on the cell type and metabolic status. One rate-limiting enzyme has been recognized for each pathway. In particular, the synthesis of NAD^+^ from NA is under the control of NA phosphoribosyltransferase (NAPRT), which catalyzes the transfer of NA to the ribose 5-phosphate moiety of phosphoribosyl pyrophosphate (PRPP), with the production of nicotinic acid mononucleotide (NAMN). Two subsequent reactions convert NAMN into NAD^+^, the first being catalyzed by nicotinamide mononucleotide adenylyl transferase (NMNAT) that adenylates NAMN to nicotinic acid adenine dinucleotide (NAAD), and the second involving NAD^+^ synthetase that transforms NAAD to NAD^+^ [1]. The NAPRT gene is frequently amplified and overexpressed in several types of tumors, including ovarian, pancreatic, liver, and colorectal cancers [2,3], which is in keeping with the higher demand of cancer cells for NAD^+^ compared to normal cells. Notably, cancers arising from normal tissues with high levels of NAPRT have a high frequency of NAPRT gene amplification and their survival fully depends on NAPRT [4]. In ovarian and pancreatic cancer cells, NAPRT silencing reduces energy status, protein synthesis, and cell size, indicating that the enzyme might represent a promising anticancer target [2]. Our group demonstrated for the first time that the enzyme is also present in a circulating, enzymatically active form [5]. The secreted enzyme markedly increases in acute inflammatory diseases such as sepsis, with a significant association between high levels of enzyme and mortality [6].

In several cell types, the NAPRT-dependent pathway occurs together with the NAD^+^ biosynthetic pathway starting from NAM and is controlled by the enzyme NAM phosphoribosyltransferase (NAMPT). NAMPT inhibition is widely recognized as an anti-inflammatory and anticancer therapy, as the enzyme is markedly overexpressed in various metabolic and inflammatory diseases, as well as in tumors [7]. Indeed, inhibitors targeting its activity have demonstrated significant anticancer activity in preclinical tumor models by depleting NAD^+^ and blocking tumor growth. However, in a clinical setting, NAMPT-specific inhibition failed as anticancer therapy [8]. One reason is that the NAMPT-dependent impairment of NAD^+^ synthesis can be circumvented by the alternative NAD^+^ biosynthetic routes, including the NAPRT-dependent pathway [2,9,10]. Indeed, cancer cells expressing high levels of NAPRT are resistant to NAMPT inhibitors and both the silencing of the gene and chemical inhibition of the enzyme overcome such resistance, suggesting that the coadministration of NAPRT and NAMPT inhibitors might improve anticancer therapies [2,11,12,13]. Altogether, these findings suggest that the inhibition of NAPRT activity might be of therapeutic potential in cancer treatment.

On the other hand, the activation of NAPRT can be regarded as a promising approach to boost intracellular NAD levels. Age-related diseases are among the conditions associated with reduced NAD function, and the restoration of NAD levels through the administration of NAD precursors or the inhibition of NAD-consuming enzymes demonstrated beneficial effects in preclinical studies [14]. Activators of the NAMPT enzyme are currently gaining great momentum because of their ability to increase NAD levels in cultured cells and in vivo studies [15,16]. Based on the evidence that NAPRT is involved in the NA-induced increase in NAD levels in different human cell lines [17], whether its pharmacological activation would raise intracellular NAD is worth investigating.

Potent NAPRT modulators are still to be identified, essentially due to the lack of a simple and fast method to assay the enzyme’s activity. Currently available methods include a radioactive-based assay, which measures the incorporation of [^14^C] NA into NAMN after separation through thin layer chromatography. This method was used in human platelet lysates to identify some NA analogues and a group of nonsteroidal anti-inflammatory drugs as inhibitors of NAPRT activity, with *K*_i_ values in the micromolar range [18,19]. More recently, our group developed a sensitive fluorometric enzyme-coupled assay to measure NAPRT activity in cell extracts and biological fluids [5]. It is a three-step assay whereby the reaction product NAMN is first converted to NAAD and then to NAD, which is finally quantified by a fluorometric cycling assay [20]. An HPLC-based method has been described for measuring the activity of purified NAPRT [21]. In this assay, after the acidification and subsequent neutralization of the reaction mixture, product formation is analyzed by using a reverse-phase C18 column, with an analysis time of about 35 min for each sample. This method was recently used to confirm the inhibitory effect on the human recombinant NAPRT of a few chemical scaffolds identified through high-throughput virtual screenings and selected due to their ability to reduce the resistance to NAMPT inhibition in a cellular setting [22,23]. All the described methods rely on multiple steps and are labor intensive and time consuming and are not suitable for the fast screening of a large set of compounds on the enzyme’s activity [5,18,19,20,21].

In this work, we describe a one-step fluorometric assay which, differently from the currently available assays, allows for the continuous measurement of NAPRT activity and can be directly performed in multiwell microplates for the simultaneous screening of different compounds. It takes advantage of the peculiar features of fluorescence technology, providing fast responses, high sensitivity, and easy handling [24]. The assay was validated by screening the effect of a set of 55 commercially available small molecules on purified human recombinant NAPRT. Molecules were selected based on NA structural features, and the screening confirmed the inhibitory effect of compounds previously identified as NAPRT inhibitors [18]. The most effective inhibitors were tested for their cytotoxicity and ability to lower NAD^+^ levels in cultured cells resistant to NAMPT inhibitors.

## 2. Results and Discussion

### 2.1. AssayDevelopment and Optimization

**Figure 1:**
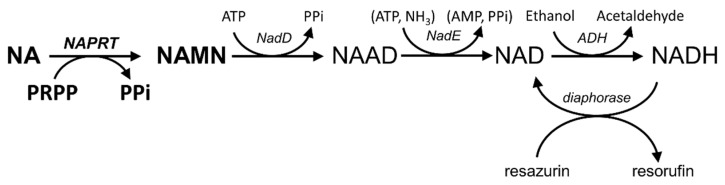
Fluorometric assays for NAPRT activity determination. The NADH assay couples NAPRT catalyzed reaction to conversion of NAMN to NADH by the sequential action of NAMN adenylyltransferase (NadD), NAD^+^ synthase (NadE), and alcohol dehydrogenase (ADH). The resazurin/diaphorase-coupled assay couples NADH formation to the cycling reduction of resazurin to resorufin by diaphorase.

**Figure 2:**
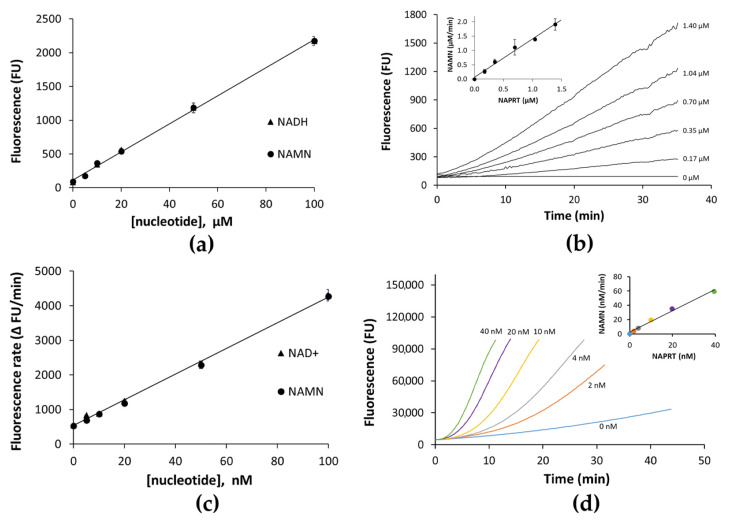
Optimization of NADH assay (**a**,**b**) and resazurin/diaphorase assay (**c**,**d**). (**a**) Stoichiometry conversion of NAMN to NADH. Various amounts of NAMN or NADH were incubated as described in the text, and fluorescence values were plotted against the nucleotides’ concentrations. (**b**) Time course of fluorescence increase in reaction mixtures containing various NAPRT concentrations. The linear correlation between NAMN formation and NAPRT concentration is shown in the inset. (**c**) Various amounts of NAMN and NAD^+^ were incubated in the resazurin/diaphorase assay mixture as described in the text, and the rates of fluorescence increase were plotted against the nucleotides’ concentrations. (**d**) Time dependence of the fluorescence increase at various NAPRT concentrations. The time course of the first derivative of each curve is reported in Supplementary Materials: Figure S1. The linear correlation between NAMN formation and NAPRT concentration is shown in the inset.

A continuous coupled fluorometric method for NAPRT activity assay was developed, whereby the formed product NAMN is converted to NADH, and NADH formation is measured fluorometrically (NADH assay). In particular, NAMN is converted to NADH through the sequential action of bacterial NAMN adenylyltransferase of the NadD family, which adenylates NAMN to NAAD, bacterial NAD^+^ synthetase (NadE) that deamidates NAAD to NAD^+^, and yeast alcohol dehydrogenase (ADH) that reduces NAD^+^ to NADH (Figure 1). The reaction mixture composition was optimized to guarantee the stoichiometric conversion of NAMN to NADH. To this end, NAPRT was substituted by NAMN or NADH at various concentrations, and fluorescence was measured after 5 min of incubation at 37 °C. As shown in Figure 2a, comparable fluorescence values were recorded, and a linear correlation was observed between fluorescence and nucleotide concentrations in the low micromolar range. The assay resulted in being sensitive down to 5 µM NAMN. To verify the assay’s suitability to determine NAPRT activity, various concentrations of the enzyme were incubated in the reaction mixture in the presence of 0.1 mM NA and 0.4 mM PRPP. As shown in Figure 2b, at any tested enzyme concentration, after an initial short lag phase, the fluorescence increased linearly with time up to at least 30 min. The amounts of NAMN produced per minute, calculated from the slopes of the linear time courses, were plotted against the enzyme concentrations (inset in Figure 2b). A linear correlation was observed between the rate of NAMN formation and the NAPRT concentrations, indicating that the ancillary system does not limit the measured activity.

It is reported that fluorescent assays that rely on 340 nm and 460 nm as excitation and emission wavelengths, respectively, can be hindered by interference from a significant fraction of compounds in screening collections. Such interference can be due to the compounds’ absorption and emission in the same wavelengths window as the assay, resulting in a false positive signal, or absorption at either the excitation or emission wavelengths, resulting in the quenching of the signal [25]. On the contrary, only a very low fraction of libraries’ compounds is optically active at wavelengths longer than 460 nm [25]. Therefore, to increase the chance of identifying NAPRT inhibitors, the assay described above was coupled to the resazurin/diaphorase system, which enabled a shift from the NADH-based blue-fluorescence readout to the resorufin-based red-shifted readout. To validate the assay, known amounts of NAMN and NAD^+^ were incubated in parallel in the reaction mixture described in the Materials and Methods section in the absence of NAPRT and its substrates. The linearity observed between the rate of fluorescence increase in the various mixtures and nucleotide concentrations indicated that diaphorase was not limiting (Figure 2c). The assay turned out to be much more sensitive than the NADH assay, being able to detect the formation of as low as 5 nM NAMN. Its suitability to determine NAPRT activity was tested by monitoring the time courses of fluorescence increase at enzyme concentrations ranging from 2 nM to 40 nM (Figure 2d). From the slopes of the first derivative of each curve plotted against the reaction time (Figure S1), the amounts of NAMN produced per minute were calculated and plotted against the enzyme concentrations (inset in Figure 2d), showing a linear dependence.

To ensure linear signal responses up to 30 min with less than 20% substrate consumption, the NADH assay and the resazurin/diaphorase assay were performed with 0.35 µM NAPRT and 2 nM NAPRT, respectively (Figure 2b,d). At 30 min of reaction, we calculated consumptions of 18% for NA and 4.5% for PRPP with the NADH assay, and 1.2% for NA and 0.3% for PRPP with the resazurin/diaphorase assay.

**Figure 3:**
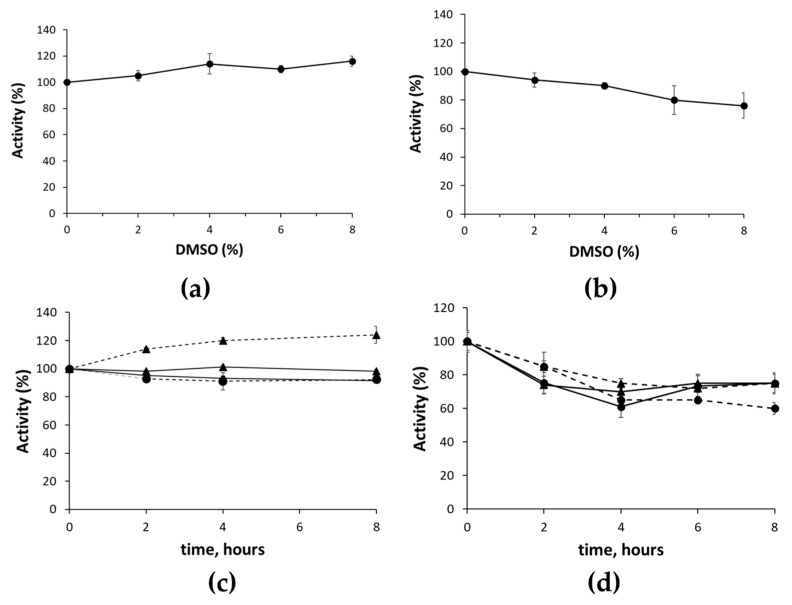
DMSO effect and reagents’ stability in NADH assay and resazurin/diaphorase assay. The effects of different amounts of DMSO on NAPRT activity was determined with NADH assay (**a**) and resazurin/diaphorase assay (**b**). Reaction mixtures, prepared as described in Materials and Methods section, contained the indicated concentrations of DMSO. The stability of the assay components in the NADH assay (**c**) and resazurin/diaphorase assay (**d**) was assessed by preincubating at 4 °C (continuous lines) or at room temperature (dotted lines) reaction mixtures containing all components except for DMSO and either NA (circle) or PRPP (triangle). At the indicated times, the reaction was initiated by adding an appropriate volume of the preincubated mixtures to wells containing DMSO (2.5% final concentration) and the missing substrate.

As most compounds in screening collections are generally soluble in DMSO solutions, the sensitivity of both assays to DMSO was determined. We found no effect on the activity of the enzyme and the ancillary system, with final concentrations of DMSO up to 8% in the NADH assay (Figure 3a), and only a slight decrease in activity was observed in the resazurin/diaphorase assay (Figure 3b).

In view of the usage of the assay in a large-scale screening, we measured the stability of the assays’ components by preincubating the reaction mixtures at room temperature or 4 °C in the absence of either one of the two substrates. At the indicated times, the reaction was started with the missing substrate. As shown in Figure 3c,d, reaction mixtures of the NADH assay were stable up to 8 h, whereas in the mixtures of the resazurin/diaphorase assay, NAPRT activity decreased to about 60% after 4 h of incubation and then remained stable up to 8 h. The latter result was likely due to the instability of some of the distinctive components of the resazurin/diaphorase assay’s mixture, i.e., FMN, resazurin, or the enzyme diaphorase. Nevertheless, the mixture appeared stable enough to enable large-sale screening.

### 2.2. Assay’s Validation

Both assays were used to screen a series of commercially available small molecules that were selected based on the structural features of NA. They also included NA analogues previously identified as NAPRT inhibitors through a radioactive-based assay performed on cell lysate [18]. Mammalian NAPRT exhibits *K*_m_ values for both substrates in the low micromolar range (from 0.5 µM to 2.5 µM for NA, and from 5 µM to 40 µM for PRPP) [26,27]. Since the NA concentration in the reaction mixture was about 40-fold the *K*_m_ value, the NA-related compounds were tested at 0.5 mM, i.e., in excess with respect to the *K*_m_, to increase the chance of identifying competitive inhibitors [28].

We screened a total of 55 compounds (listed in Table 1). Tested compounds can be classified as nicotinic acid derivatives (**1**–**8**), carboxy pyridine analogues (**25**–**27**), pyridine (**38**–**55**), pyrazine (**9**–**14**), pyridazine (**15**,**16**), pyrimidine (**28**–**33**), benzoic acid derivatives (**17**–**24**), aliphatic carboxylic acids (**34**,**35**), and other derivatives (**36**,**37**). Their effect was first tested by using the NADH assay. Compounds found to affect the NADH formation rate were also tested on the ancillary system by analyzing appropriate control mixtures, as described in the Materials and Methods section. Six compounds (**10**,**21**,**22**,**24**,**40**,**41**) out of the 55 that were tested showed optical interference at NADH fluorescence wavelength; therefore, they were tested by using the resazurin/diaphorase-coupled assay at the same substrates’ concentrations of the NADH assay. Two out of the six compounds (**21**,**22**) were found to also interfere with the resazurin/diaphorase-coupled assay, and they were tested by using the HPLC assay described in the Materials and Methods section. This assay was optimized to minimize the analysis time, which was halved when compared to the HPLC methods available in the literature [2,21]. A representative HPLC chromatogram of the NAPRT reaction mixture is shown in Figure S2.

**Figure 4:**
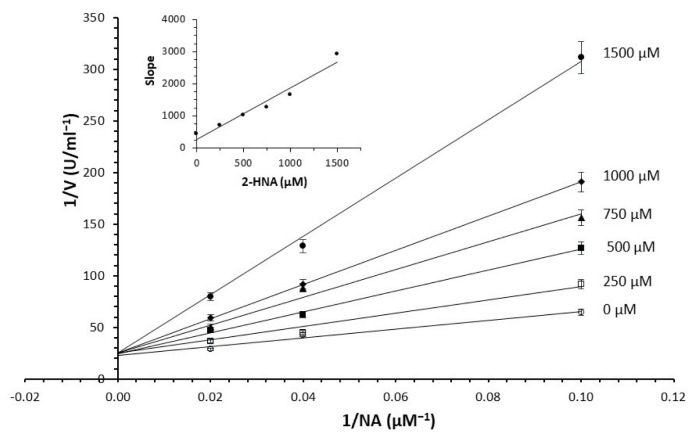
Inhibition of NAPRT by 2-hydroxynicotinic acid. Double reciprocal plot and slope replot (in the insert) of the inhibition exerted by the indicated concentrations of 2-hydroxynicotinic acid at NA ranging from 10 to 50 µM at 0.4 mM PRPP. The enzyme activity was determined by using the NADH assay. Each point is the mean of duplicate determinations.

As shown in Table 1, seven compounds exhibited more than 20% inhibitory activity against NAPRT. These compounds were further confirmed as NAPRT inhibitors by concentration-dependent inhibition assays to measure the IC_50_ value and the *K*_i_ value towards NA, as well as the inhibition mechanism. Figure 4 shows the double reciprocal plot for the *K*_i_ determination of 2-hydroxynicotinic acid that was obtained by using the NADH assay. 2-hydroxynicotinic acid was confirmed to be a competitive inhibitor of NA, with a *K*_i_ of 215 ± 5 µM [18]. The dose–response curves for IC_50_ determination and the double reciprocal plots for *K*_i_ determination of all the tested compounds are reported in Figures S3 and S4. From the kinetics performed in the absence of the inhibitor, we confirmed a *K*_m_ value for NA in the low micromolar range (15 ± 6 µM).

From the screening results summarized in Table 1, we confirmed 2-hydroxynicotinic acid (**1**), 2-amminonicotinic acid (**2**), 2-fluoronicotinic acid (**3**), pyrazine-2-carboxylic acid (**9**), and salicylic acid (**17**) as NAPRT inhibitors, with *K*_i_ ranging from 149 to 348 µM [18]. Differently from previous studies showing that these molecules were competitive inhibitors [18], we found that only the inhibition exerted by 2-hydroxynicotinic acid was competitive (Figure 4), whereas all the other molecules behaved as either noncompetitive or mixed inhibitors (Figure S4 and Table 1). This might be due to several reasons, e.g., our screening was performed on a pure enzyme, whereas previous kinetic analyses were conducted on cell lysates. Furthermore, we assayed the enzyme activity in the presence of ATP, which is required for the ancillary system being a substrate of the NadD and NadE enzymes (Figure 1). ATP has been reported to significantly affect NAPRT kinetic properties with a mechanism not yet fully elucidated. It is known that NAPRT catalyzes a facultative ATP hydrolysis involving the formation of a phosphor-histidine intermediate at the active site, resulting in the modification of the enzyme’s catalytic efficiency [29]. Indeed, when present at a 1 mM concentration, ATP increases the enzyme’s affinity for the substrates, whereas at a 3 mM concentration, it induces a negative cooperativity for both substrates [21,26,27]. It is likely that the ATP-dependent change in NAPRT kinetic behavior might affect the mechanism of inhibitors’ binding.

To make some brief consideration about the structure–activity relationship, the most effective inhibitors closely resemble the NA structure, and they all share the presence of a carboxylic group at position 3 of the pyridine ring. Moreover, the presence of small hydrogen bond acceptors such as OH, NH_2_, and F (compounds **1, 2,** and **3**) at position 2 of pyridine is required for the inhibitory effect. Moving the carboxylic acid to the other positions of the pyridyl ring, as in **25**–**27**, is detrimental for the inhibitory activity. Other pyridine derivatives, such as compounds **38**–**55**, were also inactive. Among the pyrazine derivatives, only pyrazin-2-carboxylic acid (**9**) inhibited the NAPRT enzyme with a *K*_i_ = 166 µM and a noncompetitive mechanism of inhibition. Other tested pyrazine derivatives (**10**–**14**) were inactive. Pyrimidine 2-, 4-, or 5-carboxylic acid derivatives (**29**,**31**,**28**) were very poor inhibitors (% of inhibition ≤ 20%) as well as 2-substituted pyrimidines (**30**,**32**,**33**). Pyridazine derivative bearing a COOH group in position 3 (**15**) was a mixed inhibitor with a *K*_i_ = 513 µM. Moving the COOH group from position 3 to 4 (compound **16**) drastically reduced the inhibitory activity. Among the benzoic acid derivatives **(17**–**24**), only salicylic acid (**17**) showed a noncompetitive inhibition of the enzyme with a *K*_i_ = 169 µM. Therefore, when the pyridine nucleus is replaced by a phenyl ring, the presence of a carboxylic and a hydroxylic group in position 2 seems to be required for enzyme inhibition. The aliphatic carboxylic acids (**34**,**35**) and the other derivatives (**36**,**37**) proved to be inactive.

### 2.3. Effect of NAPRT Inhibitors on OVCAR-5 Cells

**Figure 5:**
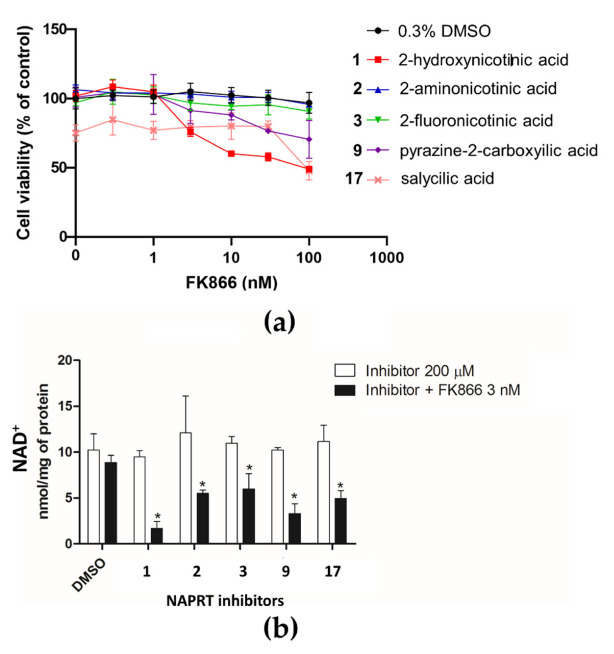
Effects of NAPRT inhibitors on OVCAR-5 cells viability and NAD^+^ content. (**a**) OVCAR-5 cells were incubated with selected compounds at 200 µM with or without the addition of different concentrations of FK866 for 72 h. Cell viability was determined using the sulforhodamine B assay. Data are mean ± SD of three experimental replicates. (**b**) OVCAR-5 cells were treated with selected compounds at 200 µM with or without 3 nM FK866. After 24 h, intracellular NAD^+^ levels were measured. Data are mean ± SD of three experimental replicates. * *p* < 0.05.

The inhibition of NAPRT by the most effective molecules was further assessed in a cellular setting. To this end, five compounds were tested on OVCAR-5 cells, known to overcome resistance to NAMPT inhibition due to the presence of NAPRT [2]. Figure 5a shows the effects of the selected compounds (**1**–**3**,**9**,**17**) on cell viability in the absence and presence of different concentrations of FK866, a potent and specific NAMPT inhibitor [30] (Figure S5).

Except for salicylic acid (**17**), the tested compounds did not modify cell viability in the absence of FK866, as cells rely on the NAMPT enzyme to produce NAD^+^ from the nicotinamide present in the culture medium. In this view, the toxicity of salicylic acid (**17**) might be due to a nonspecific activity. While 2-aminonicotinic acid (**2**) and 2-fluoronicotinic acid (**3**) did not significantly sensitize cells to FK866 toxicity, 2-hydroxynicotinic acid (**1**) and pyrazine-2-carboxylic acid (**9**) reduced cell viability when combined with FK866. A combination index analysis performed on the cell viability curves demonstrated a synergistic effect of these compounds with FK866 (Table S1).

To assess the effect of selected molecules on NAD^+^ biosynthesis, we measured intracellular NAD^+^ levels in cells treated with the compounds in the absence and presence of FK866. As shown in Figure 5b, when in the absence of FK866, the tested compounds did not affect the NAD^+^ pool, but when combined with FK866, they significantly reduced NAD^+^ levels, with 2-hydroxynicotinic acid (**1**) and pyrazine-2-carboxylic acid (**9**) being the most effective. Notably, these were the two compounds with cytotoxic activity in the cell viability experiment (Figure 5a). As shown in Figure 5, while NAD levels dropped significantly to about 15% or 30% after 72 h of incubation with 3 nM FK866 in combination with compounds **1** or **9**, respectively, cell viability was only slightly affected, being reduced to about 75% or 80%. This is in keeping with previous studies showing that in the early phase of NAD depletion, there is a decrease in cellular motility and proliferation, and only when NAD is reduced by >95%, cells lose the ability to regenerate ATP and die [31].

Our data confirm that 2-hydroxynicotinic acid is effective in sensitizing tumor cells to NAMPT inhibitors both in vitro and in vivo [2] and reveal that the replacement of the 2-hydroxynicotinic acid pyridine ring with pyrazine slightly reduces the sensitizing effect. In contrast, the substitution of the 2-hydroxynicotinic acid hydroxyl group with an amino group or a fluor atom fully abolishes the effect.

The results of the cellular experiments confirm the validity of the assays that we have developed. The proposed strategy is fast, simple, sensitive, and robust. Both ancillary enzymes NadD and NadE can be easily prepared as recombinant enzymes, and the purified proteins can be stored at −20 °C for up to one year without a significant loss of their initial catalytic activity, thus overcoming the limitation of their commercial unavailability. Both fluorometric assays are amenable to automation and miniaturization, thus reducing the cost of the screen. Altogether, these findings render the assays worthy of optimization for high throughput screening to fill the gap in targeting NAPRT.

## 3. Materials and Methods

### 3.1. NAPRT Expression and Purification

Human recombinant NAPRT was obtained as described in [21] with minor modifications. The cell pellet deriving from transformed *E. coli* BL21 (D3) cells was resuspended in one-twentieth of the original culture volume of lysis buffer (100 mM KH_2_PO_4_, pH 8.0, 300 mM KCl, 10 mM imidazole, 1mM dithiothreitol) containing 1 mM phenylmethylsulfonyl fluoride and protease inhibitor cocktail (Sigma). After two passages in a French pressure cell (18.000 psi), the crude extract was clarified by centrifugation (20 min at 20,000× *g* at 4 °C), and the supernatant was loaded onto a HisTrap FF IMAC (GE Healthcare) column (1 mL) previously equilibrated with a lysis buffer. After washing with a lysis buffer containing 30 mM imidazole, elution was performed with a linear imidazole gradient from 30 mM to 350 mM in the lysis buffer. The active fractions were pooled and desalted through a Sephadex G-25 resin (GE Healthcare) in 50 mM HEPES, pH 7.5, 50 mM KCl. The purity of the final preparation was assessed by SDS-PAGE analysis (Figure S6). Protein concentration was determined with the Bradford method [32]. The final preparation was stored at −20 °C, and after six months, the purified enzyme retained 60% of its initial catalytic activity.

### 3.2. NAPRT Activity Assays

#### 3.2.1. NADH Assay

The reaction mixture (0.2 mL) contained a 100 mM HEPES/NaOH buffer, pH 7.5; 10 mM MgCl_2_; 0.5 mg/mL bovine serum albumin; 75 mM ethanol; 30 mM semicarbazide; 4.5 mM NH_4_Cl; 0.4 U/mL nicotinate mononucleotide adenylyltransferase (NadD); 0.2 U/mL NAD^+^ synthase (NadE); 12.5 U/mL ADH; 1 mM ATP; 0.4 mM PRPP; 0. 1 mM NA; and 0.6 × 10^−3^ U/mL (0.35 µM) NAPRT. One Unit (U) NAPRT is defined as the enzyme amount catalyzing the formation of 1 µmol NAMN per minute at 37 °C. The bacterial ancillary enzymes NadD and NadE were prepared as previously described [5]. NADH fluorescence was monitored continuously at 37 °C for 30 min, using a 96-well plate in a Sinergy HT microplate reader (Bio-Tek, Winooski, VT, USA) set at excitation and emission wavelengths of 340 nm and 460 nm, respectively. The slope of the linear fit of fluorescence versus time was used to calculate the NAPRT activity by referring to an NADH standard curve, obtained by plotting the fluorescence of a known amount of NADH versus the nucleotide concentrations. Control mixtures were prepared to assay the effect of the tested compounds on the ancillary system by replacing NA, PRPP, and NAPRT with 0.5 mM NAMN and by lowering the NadD concentration down to 0.004 U/mL. These mixtures were optimized to make NadD the rate-limiting enzyme of the ancillary system, i.e., concentrations of the enzyme NadD and the NAMN substrate were chosen to guarantee a linear time course of the fluorescence increase up to 30 min.

#### 3.2.2. Resazurin/Diaphorase-Coupled Assay

For the resazurin/diaphorase assay, the reaction mixture described above also contained 4 µM flavin mononucleotide, 13 µM resazurin, and 0.46 U/mL diaphorase. The NAPRT concentration was 0.004 × 10^−3^ U/mL (2 nM). Diaphorase was previously purified through a PD MiniTrap Sephadex G-25 column (GE Healthcare) equilibrated and eluted with a 10 mM sodium phosphate buffer, pH 8.0. The increase in fluorescence due to resorufin formation in the assay was monitored continuously for 30 min, at 590 nm, after excitation at 530 nm, using a 96-well plate in the Sinergy HT microplate reader at 37 °C. Control mixtures were prepared by replacing NA, PRPP, and NAPRT with 0.1 mM NAMN and by lowering the NadD concentration down to 0.005 × 10^−3^ U/mL. To determine the NAPRT activity, the first derivative of the recorded fluorescence curve was calculated, and the slope of the corresponding curve was referred to a standard curve obtained by plotting the fluorescence rate recorded in assay mixtures containing known amounts of NAD^+^ in the absence of NAPRT, NadD, and NadE versus the dinucleotide concentrations.

#### 3.2.3. HPLC Assay

The reaction mixture contained 50 mM HEPES/NaOH, pH 7.5; 10 mM MgCl_2_; 0.5 mg/mL BSA; 1 mM ATP; 0.1 mM NA; 0.4 mM PRPP; and 1.7 × 10^−3^ U/mL (1 µM) NAPRT. The amount of enzyme was optimized to provide substrate consumption below 20% of the initial concentration after 10 min of incubation at 37 °C. In addition, withdrawals from the reaction mixture at 5 and 10 min were always performed to ensure a linear time frame. The reaction was stopped by acidification with 0.6 M HClO_4_. After 10 min on ice, samples were clarified by centrifugation for 2 min at 13,000× *g*. The supernatants were neutralized with 0.8 M K_2_CO_3_, frozen at −20 °C for about 20 min, centrifuged at 13,000× *g* for 5 min, and injected onto a Supelcosil LC-18-S column (5 µm; 250 × 4.6 mm) thermostatted at 8 °C. The column was equilibrated with 100 mM potassium phosphate, pH 6.0, containing 8 mM tetrabutylammonium hydrogen sulfate (buffer A) and eluted at a flow rate of 1 mL/min under the following gradient conditions: 3 min at 100%: buffer A; 1 min up to 90%: buffer B (buffer A plus 30% methanol); 10 min hold at 90%: buffer B; and 1 min up to 100%: buffer A, followed by re-equilibration with buffer A for 7 min. The elution was monitored at 260 nm (Shimadzu SPD-M20A), and the formed NAMN was quantified by referring to an appropriate standard.

### 3.3. Inhibitor Screening

The analyzed compounds were dissolved in 100% *v*/*v* DMSO. Few compounds were dissolved in water. The compounds were tested at a 0.5 mM concentration. No more than 2.5 % *v*/*v* DMSO was used in all assay mixtures when testing compounds prepared in DMSO. The screening was started by using the NADH assay. The protocol was as follows: 5 µL of 20 mM DMSO (or water) stock of the compound was added into a 96-weel plate followed by the addition of 5 µL of 4 mM NA. A total of 190 µL of a master mixture containing all components listed in Section 3.2.1, except for NA and the compound, was added to initiate the reaction (final volume 0.2 mL). The fluorescence increase was monitored continuously, which allowed us to easily estimate the quality of data and to identify potential artifacts (e.g., arising from compounds precipitation). Compounds found to decrease the rate of fluorescence increase were tested in control mixtures prepared as described in Section 3.2.1 to assay their effect on the ancillary enzymes. Compounds found to interfere with the ancillary system were assayed with the resazurin/diaphorase assay, with the same procedural steps of the NADH assay. One inhibitor control for each tested compound (compound and no NAPRT), as well as four positive controls (NAPRT and no compound) and four negative controls (no NAPRT and no compound) were included in every reaction plate. The percentage inhibition was calculated relative to the controls as follows:

$$\%\;\mathrm{inhibition}=\frac{\left(S_{i}-C_{i}\right)}{\left({µ}_{p}-{µ}_{n}\right)}\times 100$$

where *S_i_* represents the signal in the presence of the compound; *C_i_* is the signal of the inhibitor control; and *µ_p_* and *µ_n_* are the mean values of the signals of the positive and negative controls, respectively.

### 3.4. Kinetic Analyses

For the IC_50_ evaluation, different concentrations of each compound were tested on NAPRT activity at 0.4 mM PRPP, 0.1 mM NA by using the NADH assay. The IC_50_ value was determined by plotting the rates versus inhibitor concentration and fitting to the equation: V_i_ = V_0_/(1 + [I]/IC_50_) using GraphPad Prism 7. V_0_ and V_i_ represent initial rates in the absence and presence of the inhibitor at concentration [I], respectively.

For *K*_i_ determination, the NADH assay was used to analyze the effect of different concentrations of each compound on the enzyme’s activity at different concentrations of NA, maintaining PRPP at 0.4 mM. *K*_i_ values were calculated from the straight line deriving from the replotting of the slopes of the reciprocal plot against the concentrations of the inhibitor (secondary plot). In particular, the straight line intersects the x-axis at [I] = −*Ki* [28].

### 3.5. Cell Viability Assay

Compounds **1**, **3**, **9**, and **17** were dissolved in 100% *v*/*v* DMSO at a 100 mM final concentration. Compound **2** was dissolved in PBS at a 50 mM final concentration. The OVCAR-5 cell line was obtained from the NCI-60 panel. Cells were seeded in 96-well plates (3 × 10^3^ cells/well) and were treated with the selected compounds at a 200 µM final concentration or with 0.2% DMSO the day after, in combination with increasing concentrations of FK866, ranging from 0 to 100 nM. Treated cells were incubated at 37 °C and 5% CO_2_ for 72 h. At the end of the treatment, trichloroacetic acid (TCA) (10% final concentration) was added to each well. After 20 min at 4 °C, the wells were washed 4 times with tap water to remove TCA, and they were let to dry. Cell viability was evaluated with the sulforhodamine B assay, as previously described [33].

### 3.6. NAD^+^ Levels Measurement

The compounds were prepared as described above. OVCAR-5 cells were seeded in a 12-well plate (1 × 10^5^/well). The day after, cells were treated with combinations of each selected compound (200 µM, final concentration) and FK866 (3 nM, final concentration) and were incubated at 37 °C and 5% CO_2_. After 24 h, cells were lysed with 0.6 M perchloric acid (PCA) at 4 °C and were manually detached by a scrapper. Cell lysates were subsequently collected, transferred to new tubes, and diluted in 100 mM Na_2_HPO_4_ at pH 8. To determine the amount of NAD^+^, we utilized a sensitive cyclic assay that takes advantage of the enzymatic activity of ADH [23]. Briefly, 0.1 mL of the cycling reagent (2% (*v*/*v*) ethanol, 100 μg/mL ADH, 20 μM resazurin, 5 μg/mL diaphorase, 10 μM FMN, 10 mM nicotinamide, and 100 mM sodium phosphate, pH 8.0) were added to each well, containing 0.1 mL of each diluted sample. The increase in resorufin fluorescence (544 nm excitation, 590 nm emission) was measured every min over a 2 h period using a fluorescence plate reader (Fluostar Optima, BMG Labtechnologies GmbH, Offenburg, Germany). A NAD^+^ standard curve was always run in parallel in each assay.

## Figures and Tables

**Table 1:** Screening of selected compounds as possible NAPRT inhibitors ^1^.

ID	Structure	Compound	Inhibition (%)	IC_50_ (µM)	*K*_i_ (µM) Inhibition Mechanism
**Nicotinic acid derivatives**					
**1**	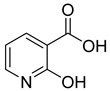	2-hydroxynicotinic acid	34	1214 ± 91	215 ± 5 competitive
**2**	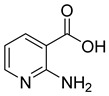	2-aminonicotinic acid	38	609 ± 69	348 ± 36 mixed
**3**	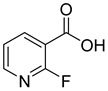	2-fluoronicotinic acid	57	351 ± 38	149 ± 14 non-competitive
**4**	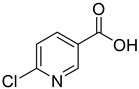	6-chloronicotinic acid	44	1259 ± 115	845 ± 69 non-competitive
**5**	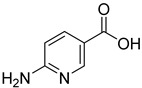	6-aminonicotinic acid	19		
**6**	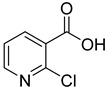	2-chloronicotinic acid	12		
**7**	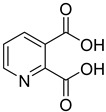	2,3-pyridine dicarboxylic acid	9		
**8**	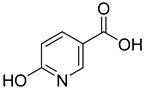	6-hydroxynicotinic acid	0		
**Pyrazine derivatives**					
**9**	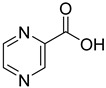	pyrazine-2-carboxylic acid	68	365 ± 36	166 ± 0.14 non-competitive
**10**	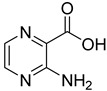	3-aminopyrazine-2-carboxylic acid	6		
**11**	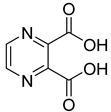	2,3-pyrazine dicarboxylic acid	0		
**12**	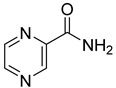	pyrazinamide	0		
**13**	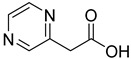	2-pyrazine acetic acid	0		
**14**	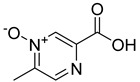	Acipimox	0		
**Pyridazine derivatives**					
**15**	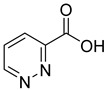	3-pyridazine carboxylic acid	30	1370 ± 74	513 ± 13 mixed
**16**	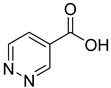	4-pyridazine carboxylic acid	18		
**Benzoic acid derivatives**					
**17**	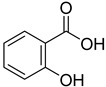	salicylic acid	58	351 ± 19	169 ± 15 non- competitive
**18**	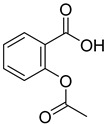	acetyl salicylic acid	11		
**19**	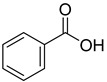	benzoic acid	8		
**20**	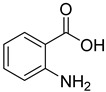	anthranilic acid	5		
**21**	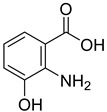	3-hydroxy anthranilic acid	5		
**22**	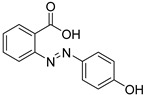	2-(4-hydroxyphenylazo) benzoic acid	5		
**23**	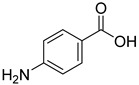	4-aminobenzoic acid	0		
**24**	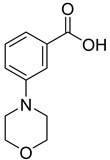	3-morpholinobenzoic acid	0		
**Carboxy pyridine analogues**					
**25**	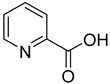	2-picolinic acid	6		
**26**	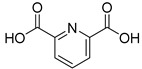	2,6-pyridine dicarboxylic acid	6		
**27**	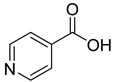	isonicotinic acid	0		
**Pyrimidine derivatives**					
**28**	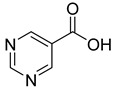	5-pyrimidine carboxylic acid	20		
**29**	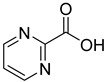	2-pyrimidine carboxylic acid	14		
**30**	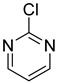	2-chloropyrimidine	12		
**31**	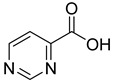	4-pyrimidine carboxylic acid	8		
**32**	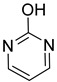	2-hydroxypyrimidine	0		
**33**	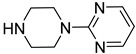	2-(1-piperazinyl)pyrimidine	0		
**Aliphatic carboxylic acids**					
**34**	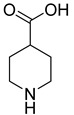	piperidine-4-carboxylic acid	0		
**35**	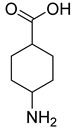	4-aminocyclohexane carboxylic acid	0		
**Other compounds**					
**36**	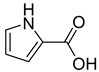	pyrrole-2-carboxylic acid	13		
**37**	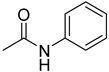	acetanilide	0		
**Pyridine derivatives**					
**38**	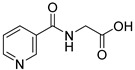	nicotinuric acid		0	
**39**	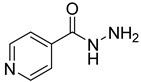	isonicotinic acid hydrazide		0	
**40**	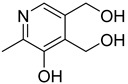	pyridoxine		0	
**41**	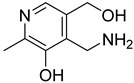	pyridoxamine		0	
**42**	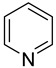	pyridine		0	
**43**	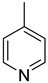	4-methylpyridine		0	
**44**	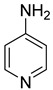	4-aminopyridine		0	
**45**	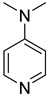	4-dimethylaminopyridine		0	
**46**	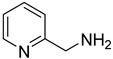	2-pyridinemethanamine		0	
**47**	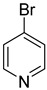	4-bromopyridine		0	
**48**	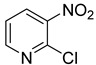	2-chloro-3-nitropyridine		0	
**49**	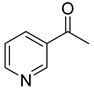	3-acetylpyridine		0	
**50**	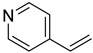	4-vinylpyridine		0	
**51**	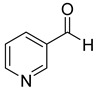	3-pyridinecarboxaldehyde		0	
**52**	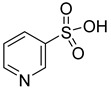	3-pyridinesulfonic acid		0	
**53**	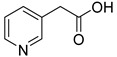	3-pyridineacetic acid		0	
**54**	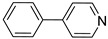	4-phenylpyridine		0	
**55**	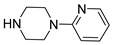	1-(2-pyridyl)piperazine		0	

^1^ NAPRT activity was determined as described in Materials and Methods section with the assay specified in the text. Percentage inhibition was determined at 0.5 mM for each compound in the presence of 0.1 mM NA and 0.4 mM PRPP; IC_50_ values were determined with the NADH assay at compound concentrations varying from 100 µM to 1 mM at 0.1 mM NA and 0.4 mM PRPP; *K*_i_ values were determined with the NADH assay at NA concentrations varying from 10 to 50 µM NA at 0.4 mM PRPP.

## Data Availability

Not applicable.

## Supplementary Materials

The following supporting information can be downloaded at https://www.mdpi.com/article/10.3390/molecules28030961/s1: Figure S1: Optimization of resazurin/diaphorase-coupled assay; Figure S2: HPLC assay of NAPRT activity; Figure S3: Dose–response curves of NAPRT inhibitors identified from the screening; Figure S4: Double-reciprocal plots and slope replots of the inhibition exerted by the selected compounds; Figure S5. Chemical structure of the NAMPT inhibitor FK866; Figure S6. SDS-PAGE analysis on 12 % polyacrylamide of the desalted IMAC pool comprising active NAPRT fractions.; Table S1: Combination index analysis.

## Sample Availability

Samples of the compounds are available from the authors.
