# Supplementary material for: A Versatile Continuous Fluorometric Enzymatic Assay for Targeting Nicotinate Phosphoribosyltransferase
Source: Molecules. 2023 Jan 18;28(3):961. doi: 10.3390/molecules28030961 (PMC9919730; doi:10.3390/molecules28030961)
Supplement: Supplementary file 1 [file molecules-28-00961-s001.zip › molecules-2131526-supplementary.pdf]

A VERSATILE CONTINUOUS FLUOROMETRIC ENZYMATIC ASSAY FOR THE SCREENING OF NICOTINATE PHOSPHORIBOSYLTRANSFERASE INHIBITORS

Gabriele Minazzato[1 ǂ], Elisa Marangoni[1 ǂ], Carlo Fortunato[1], Riccardo Petrelli [2], Loredana Cappellacci[2], Fabio Del Bello[2], Leonardo Sorci [3], Massimiliano Gasparrini [1], Francesco Piacente [4], Santina Bruzzone [4], and Nadia Raffaelli [1]*

[1] Department of Agriculture, Food and Environmental Sciences, Polytechnic University of Marche, Ancona, Italy

[2] School of Pharmacy, Chemistry Interdisciplinary Project (ChIP), University of Camerino, Italy;

[3] Division of Bioinformatics and Biochemistry, Department of Materials, Environmental Sciences and Urban Planning, Polytechnic University of Marche, Ancona, Italy;

[4]Department of Experimental Medicine, University of Genoa, Genoa, Italy

ǂ Equally contributed

* Correspondence: n.raffaelli@staff.univpm.it

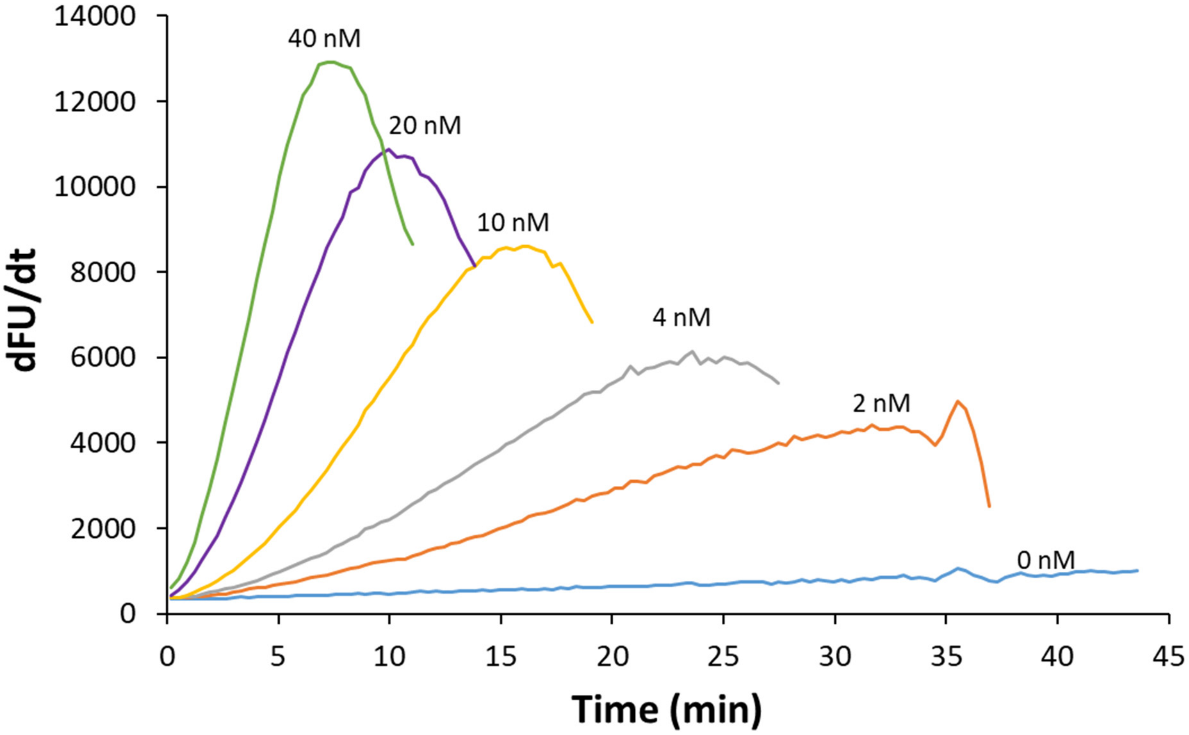

Figure S1. Optimization of resazurin/diaphorase-coupled assay. Time courses of the first derivative of the curves shown in Figure 2d. The slopes of these curves were used to calculate the amounts of NAMN produced per minute at the indicated enzyme's concentrations. Reaction mixtures were prepared as described in Materials and Methods.

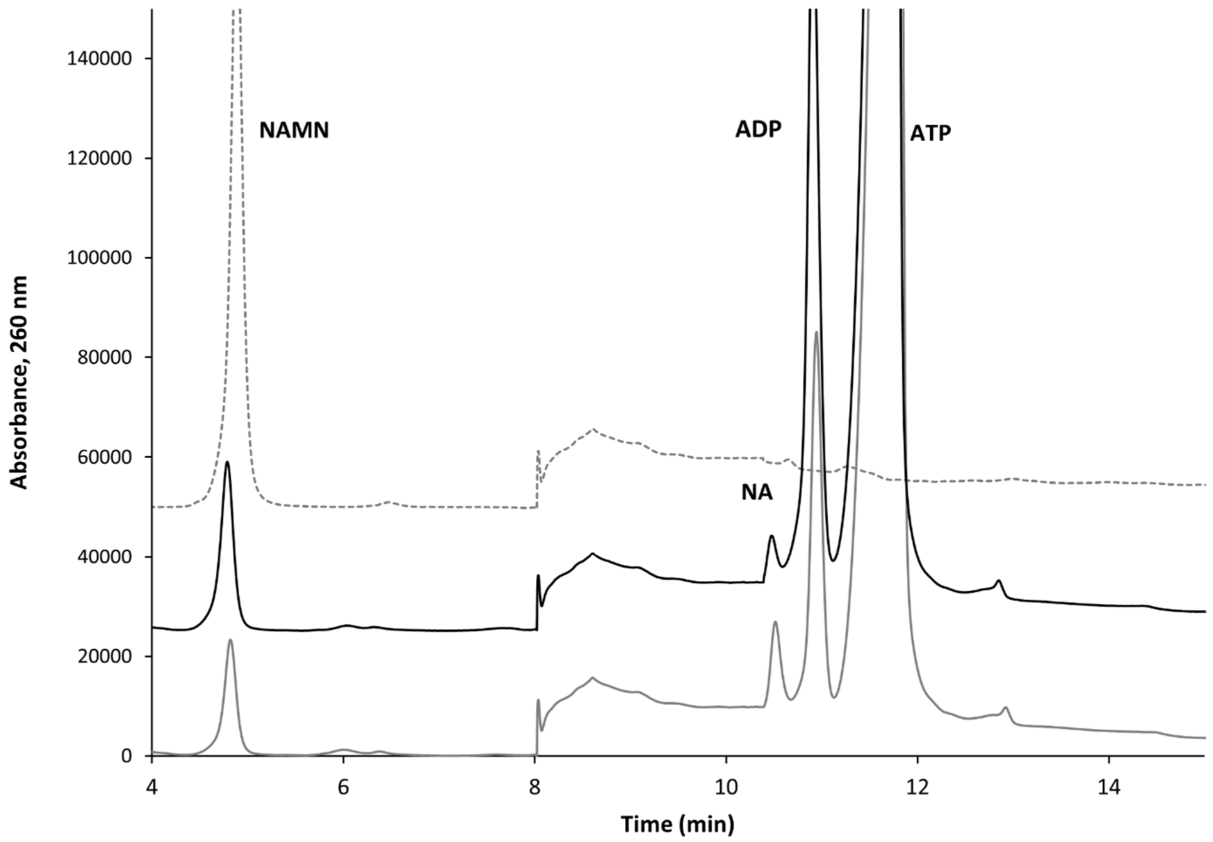

Figure S2. HPLC assay of NAPRT activity. HPLC profiles at 260 nm of the reaction mixture at 5 min (grey line) and 10 min (black line) incubation with 0.1 mM NA, 0.4 mM PRPP. A standard NAMN profile (1 nmol) is also shown (dotted line).

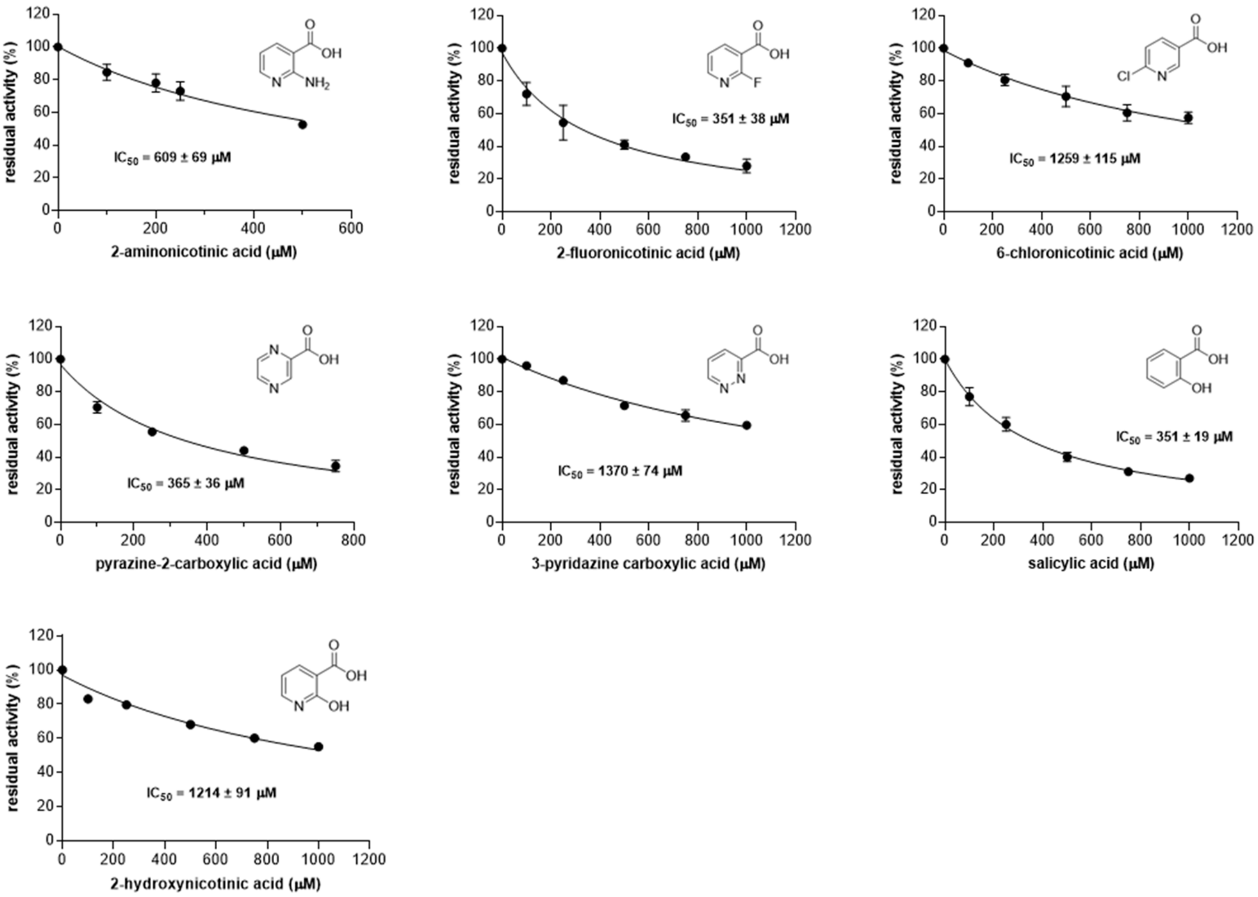

Figure S3. Dose-response curves of NAPRT inhibitors identified from the screening. The enzyme activity was determined by using the NADH assay, at compounds concentrations varying from 100 µM to 1 mM, at 0.1 mM NA and 0.4 mM PRPP. IC_{50} values were calculated as described in Materials and Methods.

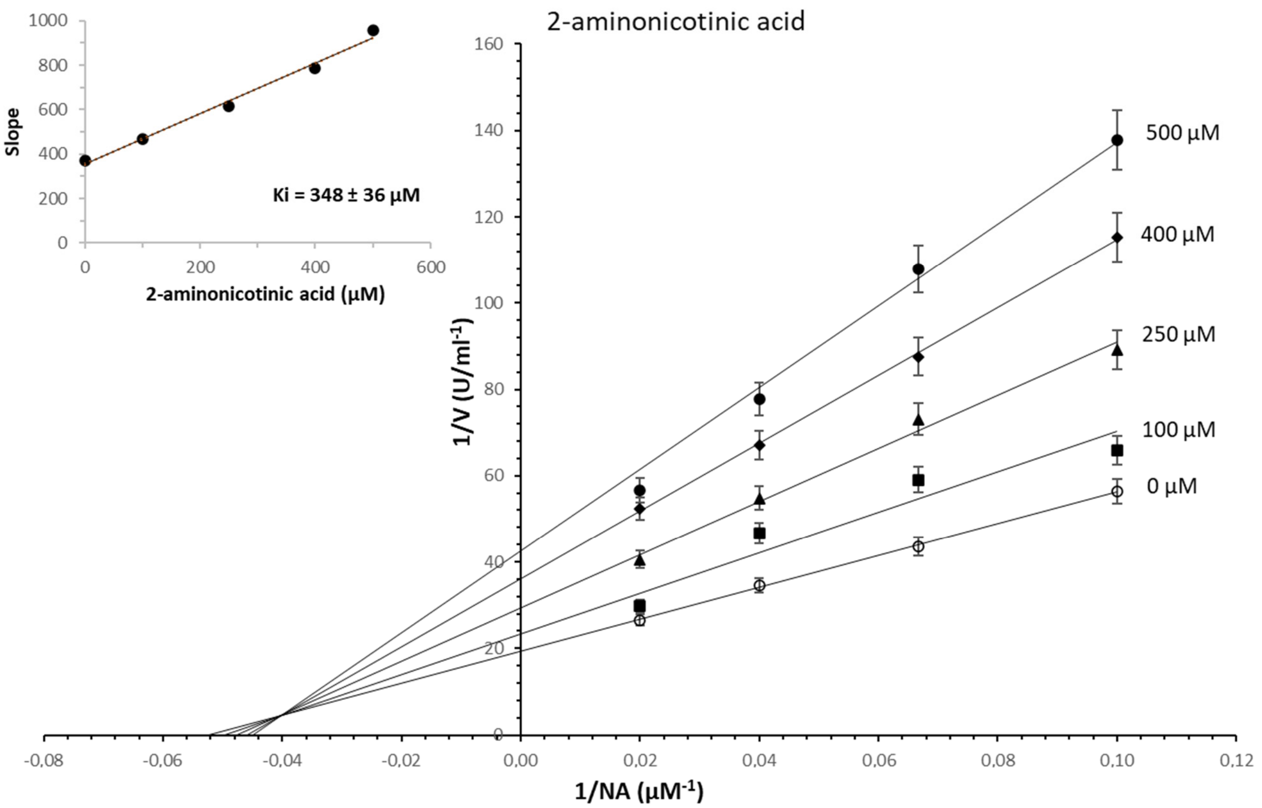
2-aminonicotinic acid
Slope
Ki = 348 ± 36 µM
2-aminonicotinic acid (µM)
1/V (U/ml-1)
1/NA (µM-1)
500 µM
400 µM
250 µM
100 µM
0 µM

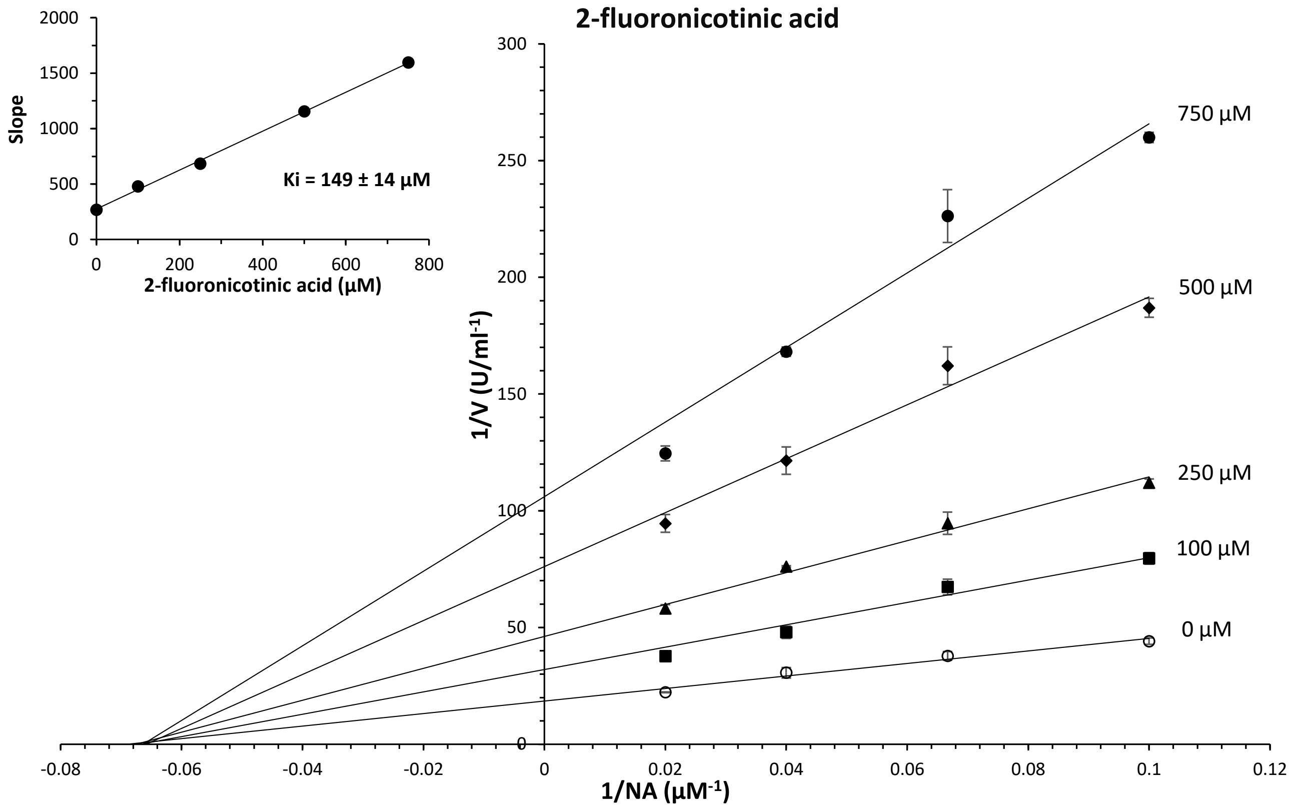
2-fluoronicotinic acid
Slope
Ki = 149 ± 14 µM
2-fluoronicotinic acid (µM)
1/V (U/ml-1)
1/NA (µM-1)
750 µM
500 µM
250 µM
100 µM
0 µM

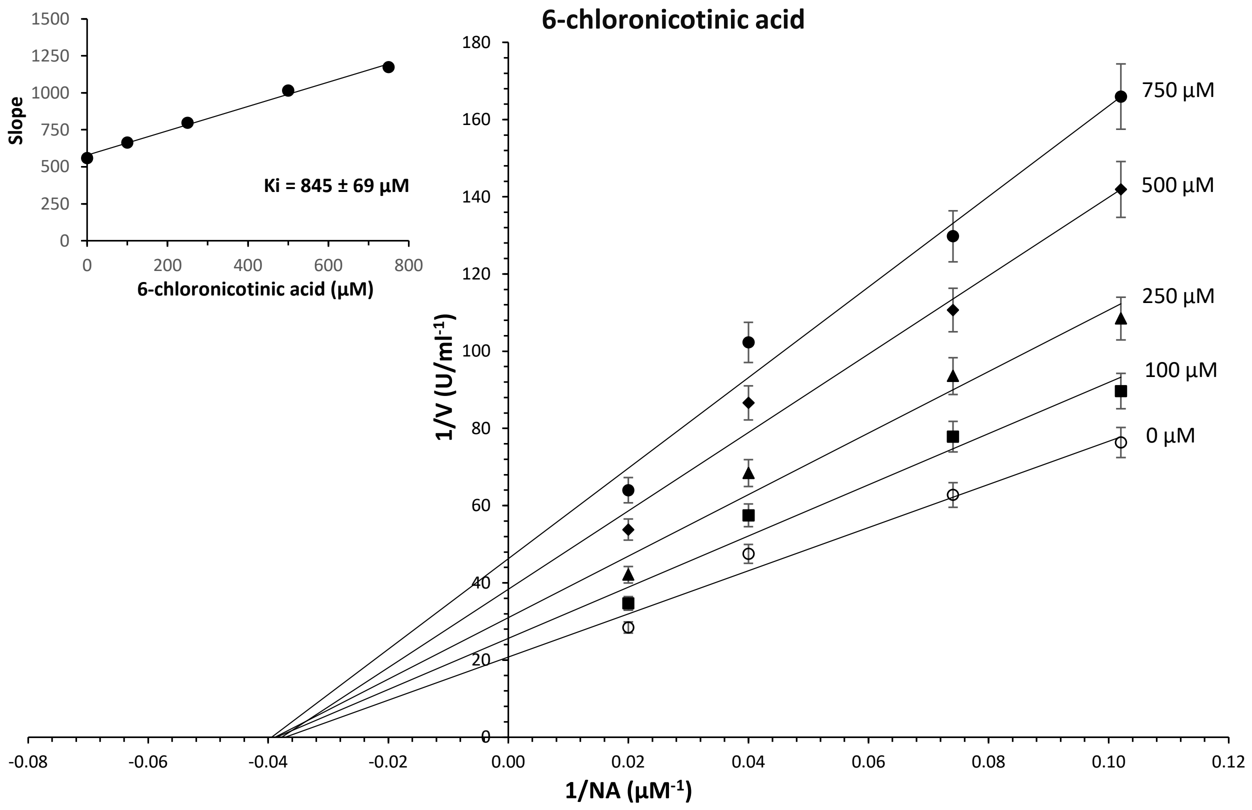
6-chloronicotinic acid
Slope
Ki = 845 ± 69 µM
6-chloronicotinic acid (µM)
750 µM
500 µM
250 µM
100 µM
0 µM
1/V (U/ml-1)
1/NA (µM-1)

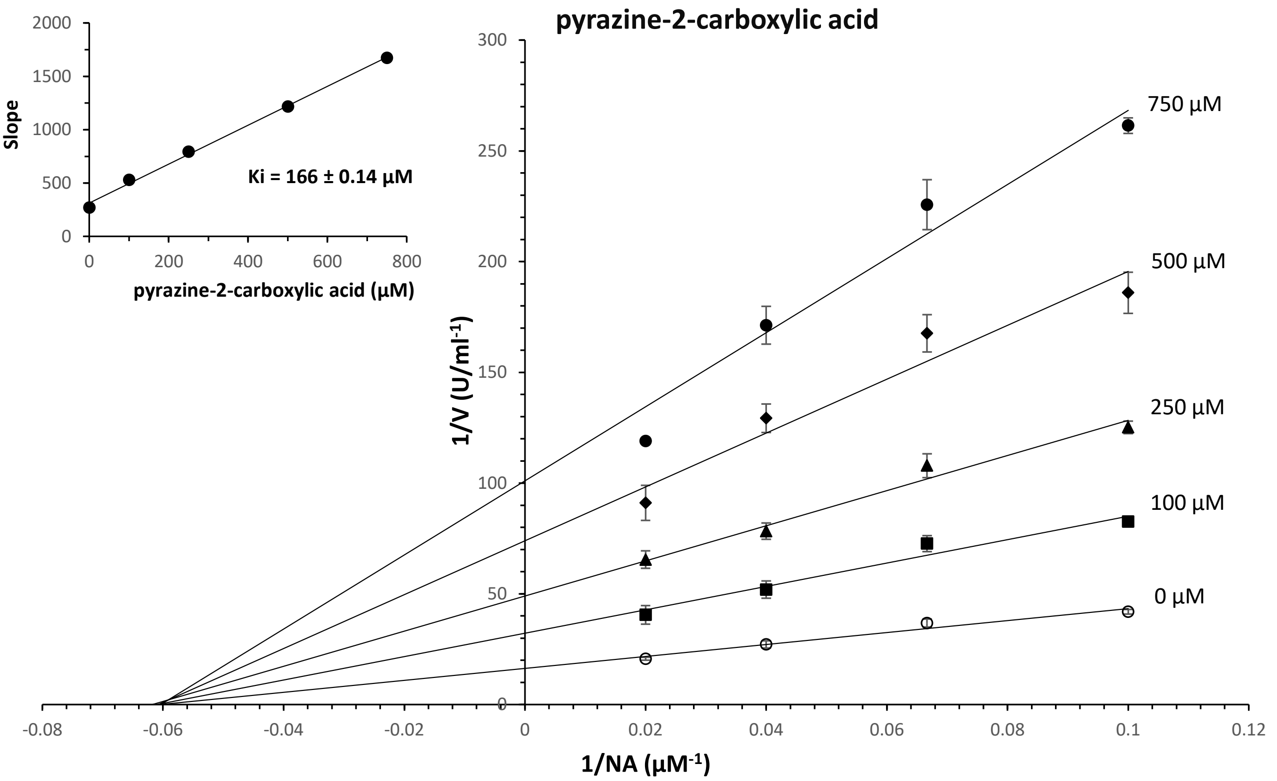
pyrazine-2-carboxylic acid
Slope
Ki = 166 ± 0.14 µM
pyrazine-2-carboxylic acid (µM)
750 µM
500 µM
250 µM
100 µM
0 µM
1/V (U/ml-1)
1/NA (µM-1)

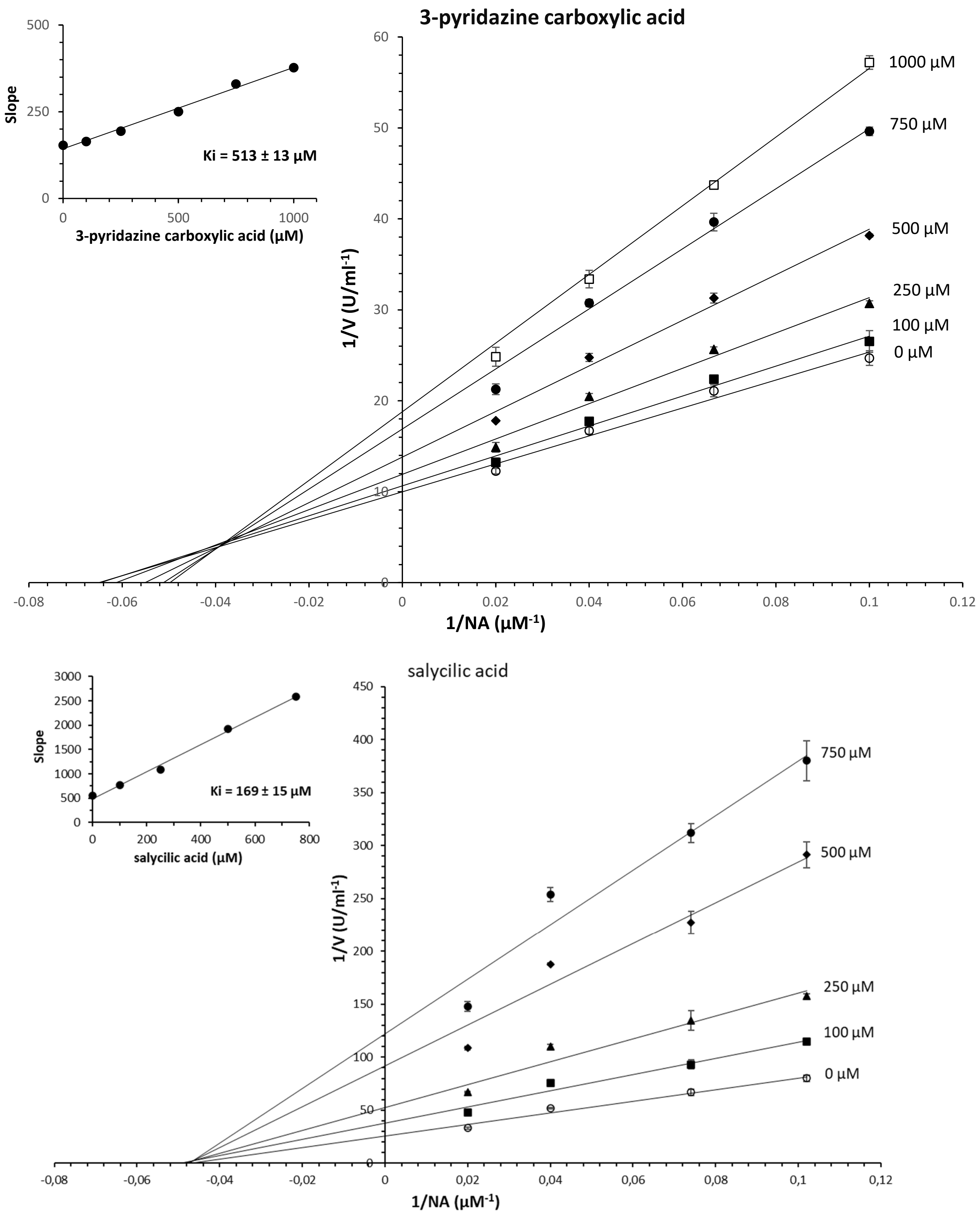

Figure S4. Double-reciprocal plots and slope replots (in the inserts) of the inhibition exerted by the selected compounds. Reaction mixtures contained the indicated concentrations of the compounds, at NA ranging from 10 to 50 µM and 0.4 mM PRPP. The enzyme activity was determined by using the NADH assay. Each point is the mean of duplicate determinations.

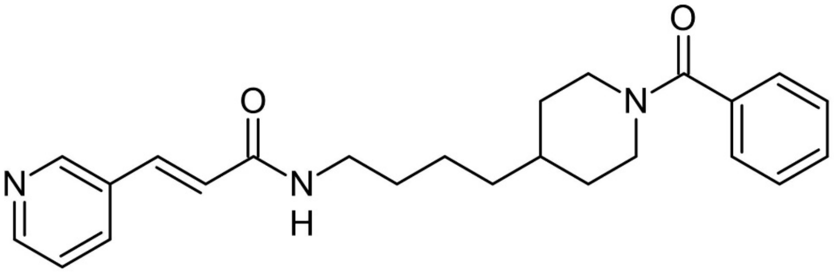

Figure S5. Chemical structure of the NAMPT inhibitor FK866

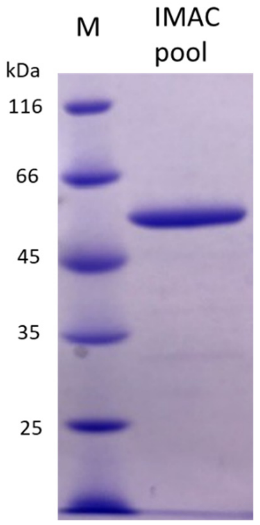

Figure S6. SDS-PAGE analysis on 12 % polyacrylamide of the desalted IMAC pool comprising active NAPRT fractions. Lane M, molecular mass standards

Table S1. Combination index analysis

	CI	
**FK866 dose (nM)**	**1**	**9**
**3**	0.51172	0.19803
**10**	0.32552	0.0673
**30**	0.33902	0.03276
**100**	0.35922	0.01211

Combination index (CI) analysis was performed on the cell viability curves obtained by treating cells with the indicated doses of FK866 and 200 µM 2-hydroxynicotinic acid (**1**) or pyrazine-2-carboxylic acid (**9**), as described in Materials and Methods. Analysis was performed using the CompuSyn software. Both compounds show a synergistic effect with FK866, resulting in CI < 1.
